# Nrf2 downregulates zymosan-induced neutrophil activation and modulates migration

**DOI:** 10.1371/journal.pone.0216465

**Published:** 2019-08-16

**Authors:** Doumet Georges Helou, Sarah Braham, Luc De Chaisemartin, Vanessa Granger, Marie-Hélène Damien, Marc Pallardy, Saadia Kerdine-Römer, Sylvie Chollet-Martin

**Affiliations:** 1 Inflammation, Chimiokines et Immunopathologie, INSERM UMR996, Univ. Paris-Sud, Université Paris-Saclay,Châtenay-Malabry, France; 2 Laboratoire d'immunologie, « Autoimmunité et Hypersensibilités », Hôpital Bichat-Claude Bernard, AP-HP, Paris, France; Medizinische Fakultat der RWTH Aachen, GERMANY

## Abstract

Polymorphonuclear neutrophils (PMNs) are the first line of defense against pathogens and their activation needs to be tightly regulated in order to limit deleterious effects. Nrf2 (Nuclear factor (erythroïd-derived 2)-like 2) transcription factor regulates oxidative stress and/or represses inflammation in various cells such as dendritic cells or macrophages. However, its involvement in PMN biology is still unclear. Using Nrf2 KO mice, we thus aimed to investigate the protective role of Nrf2 in various PMN functions such as oxidative burst, netosis, migration, cytokine production and phagocytosis, mainly in response to zymosan. We found that zymosan induced Nrf2 accumulation in PMNs leading to the upregulation of some target genes including *Hmox-1*, *Nqo1* and *Cat*. Nrf2 was able to decrease zymosan-induced PMN oxidative burst; sulforaphane-induced Nrf2 hyperexpression confirmed its implication. *Tnfα*, *Ccl3* and *Cxcl2* gene transcription was decreased in zymosan-stimulated Nrf2 KO PMNs, suggesting a role for Nrf2 in the regulation of proinflammatory cytokine production. However, Nrf2 was not involved in phagocytosis. Finally, spontaneous migration of Nrf2 KO PMNs was lower than that of WT PMNs. Moreover, in response to low concentrations of CXCL2 or CXCL12, Nrf2 KO PMN migration was decreased despite similar CXCR2 and CXCR4 expression and ATP levels in PMNs from both genotypes. Nrf2 thus seems to be required for an optimal migration. Altogether these results suggest that Nrf2 has a protective role in several PMN functions. In particular, it downregulates their activation in response to zymosan and is required for an adequate migration.

## Introduction

Polymorphonuclear neutrophils (PMNs) are the first cells to be mobilized against pathogens present in both blood and tissues. They possess a variety of killing mechanisms such as reactive oxygen species (ROS) release during oxidative burst, secretion of cytolytic enzymes and cytokines, phagocytosis and formation of neutrophil extracellular traps (NETs). They are central players in critical illness [1,2] and in chronic inflammation [3]; therefore, their activation needs to be tightly regulated to avoid tissue damage. Over the past two decades, a large number of studies have evidenced that PMNs can also behave like immune-regulatory cells [2,4]. Among the recently described mechanisms, one can emphasize the following: i) myeloperoxidase (MPO), a key PMN enzyme, can decrease mortality in a sepsis model and regulate inflammation [5,6], ii) our group and others have described PMN-induced dendritic cell modulation in particular *via* NETs [7,8], iii) NADPH oxidase 2 (NOX2) can limit inflammation in some situations [9] iiii) several inhibitory receptors and mediators have been described such as immunoglobulin-like transcript 4 or Glucocorticoïd- Induced Leucine Zipper (GILZ) [10,11].

Among the antioxidant and cytoprotective factors, nuclear factor (erythroïd-derived 2)-like 2 (Nrf2) is a transcription factor known as a master cell protector from ROS and electrophilic insult [12]. At basal state, Nrf2 is repressed by its negative regulator kelch-like ECH-associated protein 1 (Keap 1) [13]. Cellular exposure to oxidative stress or electrophiles can alter Keap1 conformation leading to nuclear translocation of Nrf2 [14]. Consequently, Nrf2 activates a battery of cytoprotective genes, such as *Nqo1* [NAD(P)H quinone oxidoreductase 1], *Hmox-1* (heme oxygenase-1) and *Cat* (catalase), all characterized by their antioxidant response element (ARE) regulatory sequence [12,15]. Typical Nrf2 activators, such as sulforaphane (SFN) and tert-butylhydroquinone (tBHQ), interact with certain cysteine residues of Keap1 implicating an electrophilic modification [16]. In addition, Nrf2 can be activated by endogenous inflammatory products such as 15-deoxy-Δ12,14-prostaglandin J2 (15d-PGJ2) and NO-derived products [17].

The Nrf2-Keap1 pathway is involved in dendritic cell (DC) and macrophage functions [18]. In particular, we have shown that Nrf2 controls cell death induced by contact sensitizers in DCs and upregulates antioxidant genes like *Gsr*, *Cat*, *Gpx* and *Nos2* controlling ROS production [19,20]. Recently, in a recent publication we described Nrf2 involvement in the control of fibrosis and autoimmunity during sclerodermia [21]. The absence of Nrf2 in immature DCs (iDCs) raises the intracellular levels of ROS and results in an enhanced co-stimulatory receptor expression associated with an increased antigen-specific CD8 T cell stimulation capacity [22]. Furthermore, the absence of Nrf2 downregulates the phagocytic functions of DCs [22] and macrophages [23,24], in particular *via* CD36 expression. In addition, Nrf2 downregulates the transcription of pro-inflammatory cytokine genes in macrophages such as *Il-6*, *Il-1β* and *Tnfα*, independently of redox control [19,25] and is involved in the expansion of suppressive myeloid-derived suppressor cells in steady state and during sepsis [26].

In contrast to DCs and macrophages, few studies have evaluated the role of Nrf2 in PMNs. It has been suggested that ROS regulation, one of the major roles of Nrf2, could contribute to the overall Nrf2 anti-inflammatory effect [24]. As Nrf2 activation is one of the ROS regulation pathways (both mitochondrial [27] and NOX2 derived-ROS [9,28,29], it can be assumed that Nrf2 could be a potential regulator of NOX2-dependent PMN functions, such as netosis. The *ex vivo* ROS production capacity of PMNs from Nrf2 Knock-out mice (Nrf2 KO) has indeed been shown to be increased during sepsis [24,30], in severe periodontitis [31] or in a model of wound healing [32].

Our aim was to better understand the role of Nrf2 in the regulation of oxidative burst and several other PMN functions. In order to do this, we chose to use zymosan as a stimulus. This insoluble cell wall preparation from *Saccharomyces cerevisiae* is known to activate phagocytes (TLR-2 and Dectin ligand) *via* the phosphorylation of p47phox subunit and rac2 activation [33]. We thus compared *in vitro*, several functions of bone marrow (BM)-derived PMNs from wild type (WT) mice and Nrf2 Knock-Out (KO) mice. We found that Nrf2 exhibited a protective role in zymosan-stimulated PMNs. Nrf2 was indeed shown to activate the transcription of the cytoprotective genes *Nqo-1*, *Hmox-1* and *Cat*, to downregulate the pro-inflammatory genes *Tnfα*, *Cxcl2* and *Ccl3* and to reduce ROS production. Furthermore, optimal migration was linked to Nrf2 expression. Interestingly, Nrf2 was not required for phagocytosis in our model. These findings could help clarify the implication of Nrf2 in clinical situations associated with PMN recruitment and/or activation. Moreover, these new findings could improve the yet to be developed comprehensive evaluation for the Nrf2-targeted therapy, as recently discussed by Cuadrado *et al*. [34].

## Material and methods

### Ethics statements

All animal studies were performed according to European Commission guidelines in compliance with French Animal Welfare Law (law n°2013–1118 from February 1^st^ 2013, article R214.89). Mice were killed for the sole purpose of collecting tibias and femurs to isolate bone-marrow. According to the French law cited above, this is not considered as an experimental procedure and no ethical approval is needed from the French Ministry of Research nor the French Ministry of Agriculture.

Tibias and femurs were collected immediately after cervical dislocation. This euthanasia procedure is in agreement with Directive 2010/63/UE of September 22 2010 annex IV and law n° 2013–1118 of February 1^st^ 2013.

### Mice

Wild-Type (WT, Nfe2l2^+/+^) and Nrf2 Knock-Out mice (Nrf2 KO, Nfe2l2^−/−^) were generated from inbred C57BL/6J background nrf2 heterozygous mice. Nrf2^−/−^ mice [35] were provided by the RIKEN BRC in accordance with a material transfer agreement (MTA) signed with Prof. S. Kerdine-Römer. The donating investigator reported that these mice were backcrossed to C57BL/6J for at least 10 generations. Mice were housed in a pathogen-free facility and handled in accordance with the principles and procedures outlined in Council Directive 2010/63/*EU*. Mice were bred side by side in ventilated racks within a specific pathogen-free facility. Age- and sex-matched mice were used at 8–14 weeks of age. Genotyping was performed by PCR using genomic DNA that was isolated from tail snips as described [35].

### BM-derived PMN isolation

PMNs were isolated from BM using the mouse neutrophil isolation Kit (Miltenyi Biotec, Bergish Glabash, Germany) according to the manufacturer’s instructions. In brief, cells were collected from the femur and tibia and resuspended in phosphate buffered saline (PBS) solution containing 0.5% bovine serum albumin (BSA) and 2 mM Ethylene diamine tetraacetic acid (EDTA). Cell suspension was then filtrated through pre-separation filters 70 μM (Miltenyi Biotec) to remove cell aggregates or large particles and ensure effective magnetic cell labeling. Cells were incubated with biotin-antibody cocktail for 10 min, washed and incubated with anti-biotin microbeads for 15 min. Finally, cell suspension was washed and applied onto a LS column placed in a magnetic field. Flow-through containing unlabeled cells representing the enriched PMN suspension, were collected and suspended in Hank’s balanced salt solution (HBSS) supplemented with 0.5% heat-inactivated fetal calf serum (FCS). Cells were kept at 4–8°C during staining and magnetic bead isolation. Cell purity and viability were assessed by flow cytometry (FACS Calibur, BD Biosciences, San Jose, USA) using antibodies against CD11b and Ly6G (BioLegend, London, UK) and was always ≥95% (S1 Fig).

### *In vitro* Nrf2 activation by sulforaphane

In some experiments, sulforaphane (SFN) (Sigma-Aldrich, St. Louis, MO, USA) was used in order to strongly activate Nrf2 in WT PMNs. Cells (1 × 10^6^/ml) were pre-incubated with SFN 1 μM for 4 h at 37 °C with 5% CO_2_. As SFN can be toxic in some conditions, we checked that none of the tested SFN concentrations (1 and 5 μM) were either toxic or induced apoptosis within 4 h, using the Annexin V/7-amino-actinomycin (AnnV/7AAD) counterstaining (BioLegend) followed by flow cytometry (FACS Calibur). Cells positive for Annexin V and negative for 7-AAD were considered as apoptotic cells (consistently under 3%), whereas double positive cells were considered as necrotic cells (consistently under 8%) (S2 Fig).

### Nrf2 quantification using flow cytometry

PMNs (1 × 10^6^/ml) from WT mice were stimulated with SFN (1 μM), or with 5 μg/ml of zymosan A (Sigma-Aldrich, suspended uniformly in HBSS) for 4 h at 37 °C with 5% CO_2_. After fixation and nuclear permeabilization using a commercial kit (ThermoFisher Scientific, California, USA), cells were incubated with a rabbit monoclonal anti-Nrf2 antibody detecting nuclear and cytoplasmic Nrf2 (ab62352, Abcam, Cambridge, UK), and then with a goat anti-rabbit Alexa Fluor 488 IgG (ThermoFisher Scientific). IgG antibody isotype control was used as a negative control (BD Biosciences). Intracellular total Nrf2 expression was quantified in all the samples using an Attune NxT flow cytometer (Thermofisher Scientific). In some experiments, whole BM cells were stained directly without PMN isolation; in that case, the anti-Ly6G antibody was used to identify PMNs.

### Quantitative reverse transcription-polymerase chain reaction (RT-qPCR)

PMNs (1 × 10^6^/ml) from WT and Nrf2 KO mice were stimulated with SFN (1 μM), or with zymosan (5 μg/ml) for 4 h at 37 °C with 5% CO_2_. Total RNA was extracted after PMN lysis with RNA-PLUS reagent (MP Biomedicals, Santa Ana, CA, USA). Total RNA pellets were resuspended in RNAse-free water and quantified by spectrophotometry. First-strand cDNA was synthesized from total RNA on a thermocycler (Biometra, Göttingen, Germany). The reaction used 1 μg of total RNA, a dNTP mixture (containing 25 mM dATP, dGTP, dCTP, and dTTP) and 50 μM oligo (dT) primers (MWG Biotech, Ebersberg, Germany). Reverse transcription was performed in 1× AMV reverse transcriptase reaction buffer (Promega, Charbonnières-les-Bains, France), with RNase inhibitor (RNasine; Promega) at 40 U/μl, AMV reverse transcriptase (Promega) at 10 U/μl, and RNase-free water, to a final volume of 10 μl. A control without reverse transcriptase was used to confirm the absence of DNA contamination. RT-qPCR was performed with SYBR Green technology on a CFX96 system (Bio-Rad, Marnes-la-Coquette, France). Each reaction mix consisted of 1:50 diluted cDNA in a 4 μl final volume of nuclease-free water; 0.5 μM of each forward and reverse primer for *Hmox-1*, *Nqo1*, *Cat*, *Cxcl1*, *Cxcl2*, *Ccl3*, *Il-6*, *Tnfα*, *Il-1β*, *Gapdh*, *β-actin*; and Sso Advanced Supermix (Bio-Rad) in a total reaction volume of 10 μl. The following specific primers were used (forward and reverse, respectively): *Hmox-1*: 5’-AGG GTC AGG TGT CCA GAG AA-3’ and 5’-CTT CCA GGG CCG TGT AGA TA-3’; *Nqo1*: 5’-ACG GGG ACA TGA ACG TCA TTC T-3’ and 5’-AGT GTG GCC AAT GCT GTA AAC C-3’; *Cat*: 5’-GTG GTT TTC ACT GAC GAG ATG GCA-3’ and 5’-TCG TGG GTG ACC TCA AAG TAT CC-3’; *Cxcl1*: 5’-GGC CCC ACT GCA CCC AAA CC-3’ and 5’-CCG AGC GAG ACG AGA CCA GGA GA-3’; *Cxcl2*: 5’-CTC TCA AGG GCG GTC AAA AAG TT-3’ and 5’-TCA GAC AGC GAG GCA CAT CAG GTA-3’; *Ccl3*: 5’-ACC ACT GCC CTT GCT-3’ and 5’-TGG AAT CTT CCG GCT-3’; *Il-6*: 5’-AGT TGC CTT CTT GGG ACT GA-3’ and 5’-CAG AAT TGC CAT TGC ACA AC-3’; *Tnfα*: 5’-CAC CAC GCT CTT CTG TCT AC-3’; *Il-1β*: *5’-ACA GCA GCA CAT* CAA-3’ and 5’-GCA GGT TAT CAT CAT-3’; *Gapdh*: 5’-TGC ACC ACC AAC TGC TTA G-3’ and 5’-GAT CCA GGG ATG ATG TTC-3’; *β-actin*: 5’-CCT TCT TGG GTA TGG AAT C-3’ and 5’-AGG TCT TTA CGG ATG TCA AC-3’.

After 30 s at 95°C for Sso7dfusion polymerase activation, amplification was allowed to proceed for 44 cycles, each consisting of denaturation at 95°C for 5 s and annealing/extension at 60°C for 5 s. Eightfold serial dilutions of mixed cDNA (from different samples) were analyzed for each target gene, enabling us to construct linear standard curves from which the efficiency (E) of each PCR run was evaluated. SYBR green fluorescence was detected at the end of each elongation cycle, after which a melting curve was constructed to confirm the specificity of the PCR products. Quantification was performed with CFX Manager Software (Bio-Rad), and data were analyzed by the ΔΔCt method. Ratios were calculated as the geometric mean of (1 + E) ^− ΔΔCt^, where E is the efficiency and ΔΔCt is the target gene expression of treated cells compared with normal levels in untreated cells, with correction for the expression of the reference genes *β-actin* and *Gapdh*. Results are expressed as the fold factor increase (i.e., ratio of (1 + E)^− ΔΔCt^ of treated cells/(1 + E) ^− ΔΔCt^ of untreated WT cells).

### Analysis of ROS production

The sum of intra- and extra-cellular ROS produced was quantified by luminol (5-amino-2,3-dihydro-1,4-phthalazindione)-amplified chemiluminescence assay. Isolated WT and Nrf2 KO PMNs were seeded at 1 × 10^5^/well in a white flat bottom 96-well plate (Costar, Kennebunk ME, USA) and treated with 0.06 mM luminol (Sigma-Aldrich). Cells were then stimulated with increasing concentrations of zymosan (1, 5 and 10 μg/ml). In some experiments, a preincubation with SFN 1 μM for 4 h was carried out before cell stimulation, allowing for optimal Nrf2 expression. PMNs without stimulation were used as controls. ROS-dependent chemiluminescence was analyzed immediately using a multimode microplate reader (TristarTM LB941 Berthold, Bad Wildbad, Germany). ROS release was monitored for 60 min every 30 sec at 37°C. All samples were tested in triplicate. The area under the curve (AUC) of each sample was calculated.

### Induction and quantification of neutrophil extracellular traps (NETs)

NET analysis was performed as previously described [36]. Briefly, staining with the non-cell-permeable DNA dye SYTOXgreen (Invitrogen, Carlsbad, USA) was used to evaluate the kinetics of extracellular DNA release. PMNs (1 × 10^5^) in HBSS medium were seeded to a Cellstar black 96-well plate (Greiner Bio-One, Frickenhausen, Germany). SYTOXgreen (5 μM) was added 20 min before PMN stimulation or not with PMA 100 nM or zymosan 50 μg/ml. The fluorescence of NET-bound SYTOXgreen (excitation: 488 nm, emission: 510 nm) was analyzed for a period of 3 h every 15 min at 37°C using LB 941 Multimode reader TriStar. NET release was calculated as the difference between the mean relative fluorescence unit (RFU) at time 15 min and the RFU at time 180 min.

### Phagocytosis analysis

Phagocytosis analysis was performed as per supplier’s instructions using pHrodo Red zymosan BioParticles (Life Technologies, Carlsbad, USA) conjugate for phagocytosis. Briefly, isolated WT and Nrf2 KO PMNs (1 × 10^6^/ml) were seeded in duplicate wells, pretreated or not with cytochalasin D (20 μM) (Sigma-Aldrich) at 37 °C with 5% CO_2_ for 30 min and then incubated for 90 min in the dark, alone or with increasing concentrations of zymosan bioparticles (5–50 μg/ml). Using flow cytometry (FACS Calibur), phagocytosis was quantified by the increase in particle fluorescence in acidic compartments. Cells were subjected to a one-color analysis (FL-3, PerCP) for the percent of zymosan positive cells.

### Chemotaxis assay

Chemotaxis assay was performed on freshly isolated WT and Nrf2 KO PMNs using transwell migration assay, as previously described [37]. Briefly, PMNs (1 × 10^6^ cells) were added to the upper chamber of Transwell filters (3 μm pore diameter, Costar). These chambers were placed in 24-well cell culture plates containing 600 μL assay buffer without chemoattractant or with N-Formylmethionyl-leucyl-phenylalanine (fMLP, 1 μM) (Sigma-Aldrich), increasing concentrations of CXCL2 (0.5–200 nM) and increasing concentrations of CXCL12 (50–400 nM) (both from BioLegend). In some experiments, cells were preincubated with AMD3100 octahydrochloride 50 nM (Sigma-Aldrich) for 30 min before being placed in the upper Transwell chamber to confirm the specificity of CXCR4-dependent migration. Chambers were then incubated for 60 min at 37°C with 5% CO2 and the cells that had migrated to the bottom chamber were recovered and stained with antibodies against Ly6G (PercP. Cy5.5) and CD11b (FITC) (both from BioLegend) for flow cytometry analysis (FACS Calibur). Chemotactic indexes were then calculated by dividing the number of PMNs counted in chemokine-stimulated wells by the number of PMNs counted in filter-free wells (input well without any chemokine).

### Quantification of receptor expression

The expression of TLR2, CXCR2 and CXCR4 on freshly isolated WT and KO PMNs was evaluated using flow cytometry. First, PMNs were incubated with anti-FcR antibody (anti-CD16/CD32, BD Biosciences) at 4°C for 15 min. Then, cells were washed and incubated in the dark with antibodies against Ly6G, TLR2, CXCR2 and CXCR4 or with corresponding isotypes (all from BioLegend). Ly6G positive cells were subjected to a double-color analysis to measure the mean fluorescence intensity (MFI) for receptors.

### ATP measurement

The level of ATP was measured in freshly isolated PMNs using Luminescence ATP detection Assay Kit (Abcam) following the manufacturer’s instructions. Isolated resting WT and Nrf2 KO PMNs were seeded at 1 × 10^5^/well in a white flat bottom 96-well plate (Costar) without any stimulation. Samples were tested in triplicate. Luminescence was quantified using the multimode microplate reader (TristarTM LB941 Berthold) and then converted to ATP concentration (in μM) using standard curve.

### Statistical analysis

Nonparametric analyses were performed using GraphPad Prism software: the Mann Whitney test was used to compare two independent groups and the Kruskal-Wallis test for more than two independent groups. Data are expressed as means ± SEM. P<0.05 was considered to denote statistical signficance.

## Results

### Nrf2 is inducible in BM-derived WT PMNs and triggers the activation of its target genes

**Fig 1:**
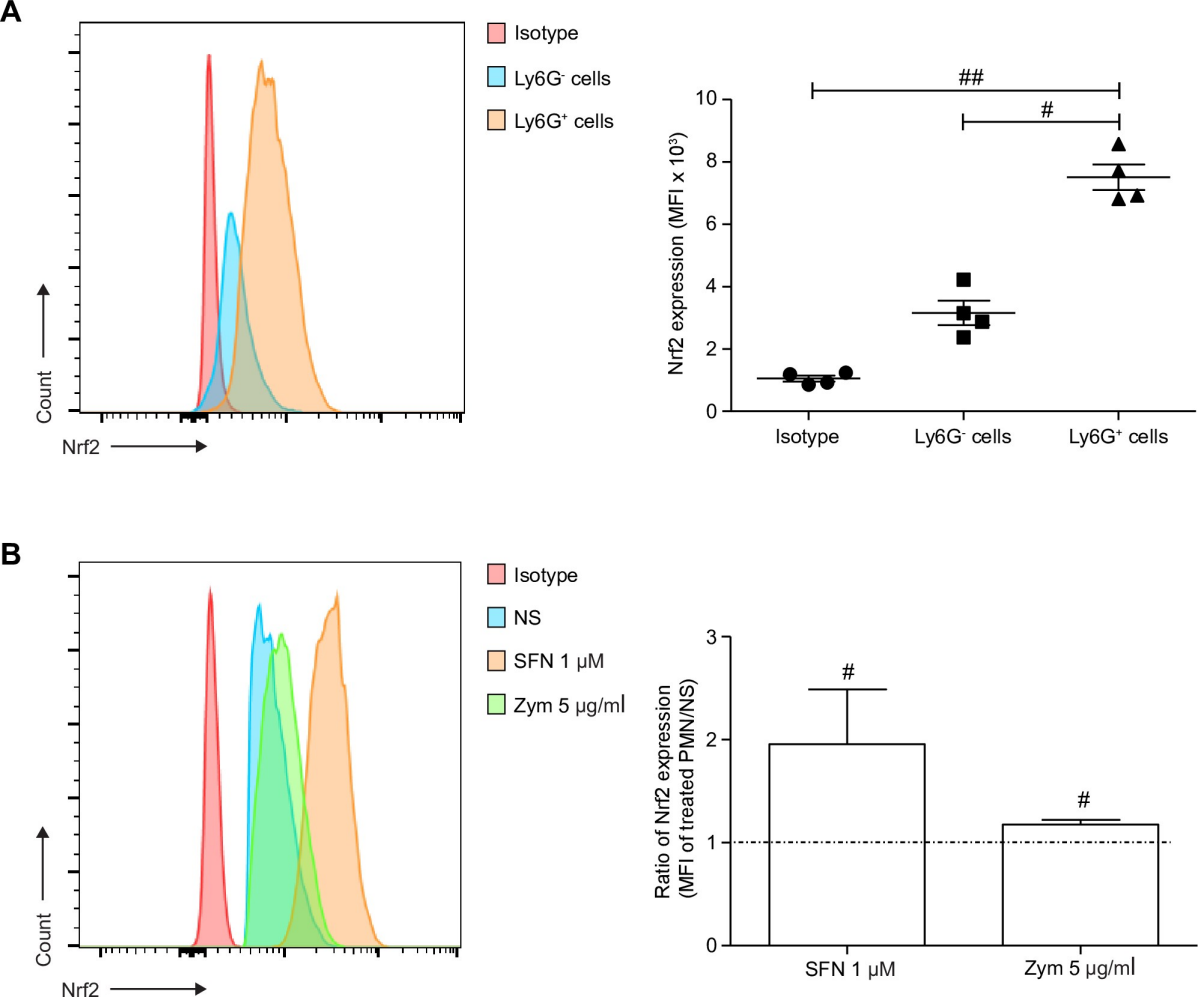
Nrf2 endogenous expression is high in PMNs and increases in response to SFN and zymosan. Freshly obtained BM cells were stained with anti-Ly6G and rabbit anti-Nrf2 IgG or with corresponding isotype, then with goat anti-rabbit Alexa Fluor 488 Ab. Isolated PMNs were incubated for 4 h alone (NS), with 1 μM of SFN before staining or with zymosan 5 μg/ml. (A) Endogenous Nrf2 was quantified in BM cells using intracellular staining followed by flow cytometry. PMNs were identified as Ly6G^+^, while Ly6G^-^ cells represents the rest of BM cells. Nrf2 expression was quantified as the mean fluorescence intensity (MFI) and compared between Ly6G^+^ and Ly6G^-^ cells (#p<0.05 and ##p<0.01, Kruskal-Wallis test)**.** (B) Nrf2 accumulation in isolated PMNs after incubation with SFN 1 μM or zymosan 5 μg/ml, was measured using flow cytometry. The ratio of Nrf2 expression was calculated by dividing the MFI of treated PMNs by the MFI of untreated PMNs (NS). Ratios greater than 1 indicate Nrf2 accumulation in stimulated PMNs (Mann-Whitney test). Results are the mean ± SEM of samples from 4 independent experiments, n = 4.

As a first step, we assessed Nrf2 expression in WT PMNs since Nrf2 is an ubiquitous transcription factor, constantly ubiquitinilated by its cytosolic repressor Keap1 [38]. In a first set of experiments, BM cells from WT mice were stained directly after filtration in order to quantify Nrf2 endogenous expression in PMNs, in comparison to other BM cells. As expected, Nrf2 was expressed in almost all BM cells; interestingly, its expression in PMNs (identified as Ly6G^+^ cells) was at least 2-fold higher than in the other BM cells (Fig 1A).

As Nrf2 was highly expressed in WT PMNs, we evaluated its accumulation in response to SFN (a well-known Nrf2 activator) used as a positive control stimulus, or to zymosan a strong PMN stimulus. Freshly isolated BM-derived WT PMNs were thus incubated alone, with SFN or with zymosan, for 4 h. Using flow cytometry, we evidenced that zymosan as well as SFN 1 μM were able to significantly increase the intracellular staining of Nrf2 in WT PMNs, indicating its accumulation (Fig 1B).

**Fig 2:**
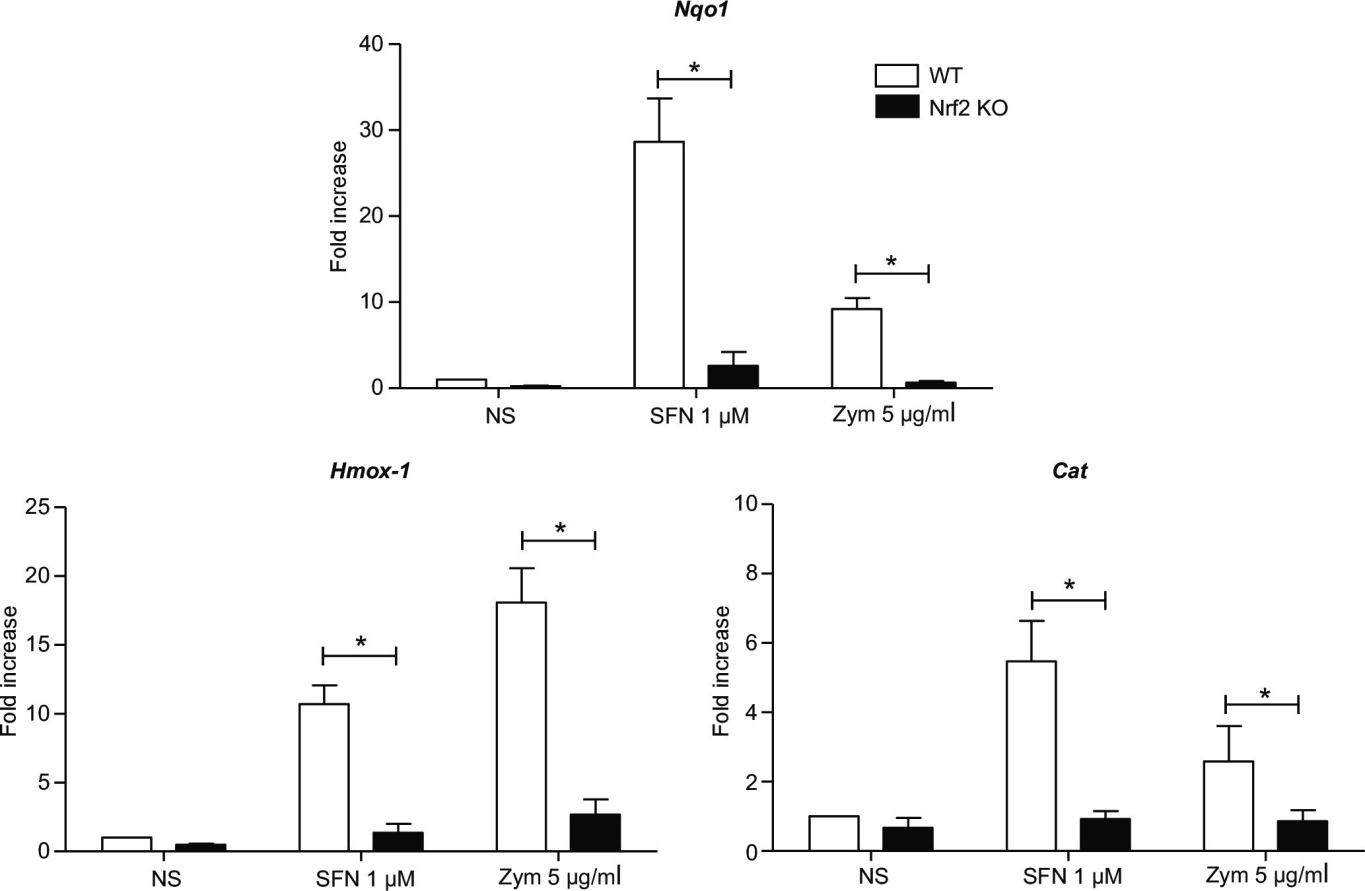
Nrf2 upregulates the transcription of 3 main target genes in response to SFN and zymosan. WT and Nrf2 KO PMNs were incubated or not (NS) with SFN 1μM or zymosan 5 μg/ml for 4 h. mRNA expression of *Nqo1*, *Hmox-1* and *Cat* was quantified using RT-qPCR. Results are expressed as fold increase normalized to WT NS and corrected by the expression of the housekeeping genes *β-actin* and *gapdh* in all RT-qPCR experiments. Results are the mean ± SEM of samples from 4 independent experiments, n = 4 (* indicates a significant difference between WT and Nrf2 KO PMNs throughout the study, *p<0.05, Mann-Whitney test).

Finally, the transcription of three downstream target genes of Nrf2 (*Nqo1*, *Hmox-1* and *Cat*) was evaluated in WT and Nrf2 KO PMNs, in response to SFN and zymosan. In accordance with flow cytometry results, SFN and zymosan induced the transcription of *Nqo1*, *Hmox-1* and *Cat* in WT PMNs (Fig 2).

Taken together, these results strongly suggest that like SFN, zymosan can induce Nrf2 accumulation and activate its target genes in WT PMNs. As expected, no Nrf2 target gene transcription was observed in Nrf2 KO PMNs.

### Nrf2 activation in PMNs participates in the regulation of zymosan-induced oxidative burst

**Fig 3:**
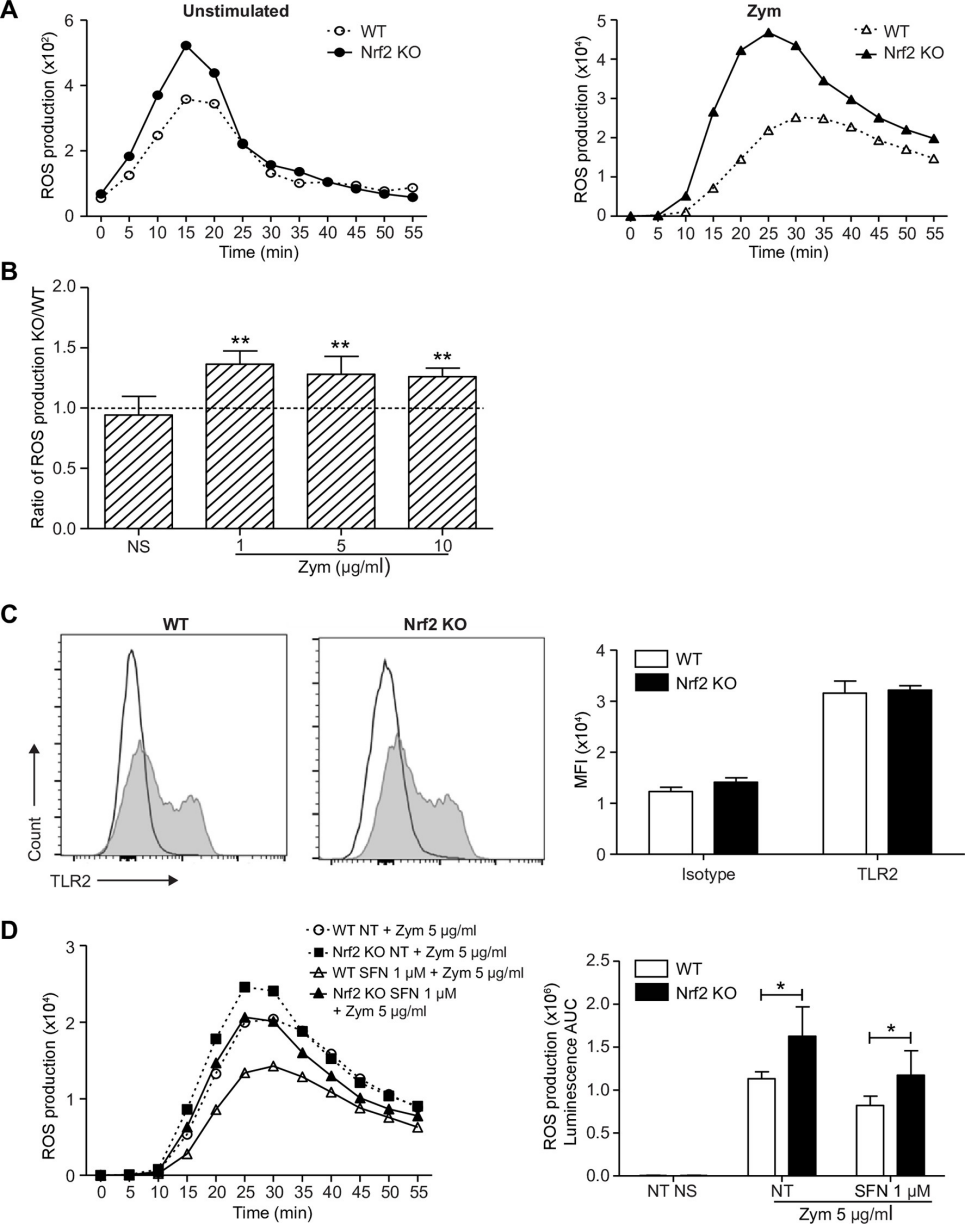
Nrf2 activation decreases PMN ROS production in response to zymosan. WT and Nrf2 KO PMNs were stimulated or not with increasing concentrations of zymosan. The sum of intra- and extracellular ROS was monitored for 60 min by the luminol-amplified chemiluminescence assay. (A) Graphs from one representative experiment show the kinetic of ROS production in unstimulated PMNs (left panel) or in response to zymosan 10 μg/ml (right panel). (B) The area under curve (AUC) from the kinetic curves of 6 independent experiments was used to calculate the ratio of ROS production (KO AUC/WT AUC). Ratios greater than 1 correspond to higher ROS production in KO PMNs (**p<0.01, Mann-Whitney test). (C) The expression of TLR2 was assessed on freshly isolated WT and Nrf2 KO PMNs (grey filled curves) in comparison to corresponding isotypes (black unfilled curves). The associated graph represents the mean ± SEM of TLR2 MFI, n = 4. (D) To ensure an important Nrf2 accumulation, PMNs were pretreated or not (NT) with SFN 1 μM for 4 h then stimulated with 5 μg/ml of zymosan. ROS production was then measured for 60 min by the luminol-amplified chemiluminescence assay. Graphs show the kinetics of ROS production from one representative experiment (left panel) and the mean ± SEM of AUC from the kinetic curves of 6 independent experiments (right panel, n = 6; *p<0.05, Mann-Whitney test).

As Nrf2 is inducible in PMNs, we aimed to study its implication in oxidative burst. First, a luminol-amplified chemiluminescence assay was used to evaluate the role of Nrf2 on both intra- and extracellular ROS production in response to zymosan. In comparison to WT, PMNs from Nrf2 KO mice exhibit a shift in the kinetics of ROS production, characterized by an early peak at 25 min following stimulation with zymosan (Fig 3A). In response to increasing concentrations of zymosan (1, 5 and 10 μg/ml), we quantified a significantly higher production of ROS in Nrf2 KO PMNs than in WT PMNs suggesting an implication of Nrf2 in the regulation of ROS production (Fig 3A and 3B). As zymosan-induced activation is dependent on TLR2 activation, we wondered if Nrf2 KO PMNs displayed a higher basal expression of TLR2. Flow cytometric analysis showed that PMNs from both genotypes displayed a similar expression of TLR2 (Fig 3C). Similar results were observed with dectin-1 expression, another receptor involved in zymosan-induced PMN activation (data not shown).

Next, the effect of a significant Nrf2 accumulation on the regulation of ROS production was assessed. WT and Nrf2 KO PMNs were thus pre-treated or not with 1 μM of SFN at 37°C for 4 h, before stimulation with zymosan at 5 μg/ml. As shown in Fig 3D, SFN-preincubation more significantly decreased ROS production in WT PMNs than in Nrf2 KO PMNs suggesting that Nrf2 activation decreases ROS production in PMNs.

As netosis can be related to ROS production in most conditions, we wanted to examine the possibility that Nrf2 might also modulate this function, using the SYTOXgreen assay. We found that PMA-induced extracellular DNA release was similar in WT and KO PMNs, as zymosan failed to induce netosis in PMNs from WT and Nrf2 KO mice (S3 Fig). We could thus rule out a potential effect for Nrf2 in these conditions.

Altogether, these results provide evidence that Nrf2 activation participates in the regulation of ROS production in BM-derived PMNs in response to zymosan independently of TLR2 expression level.

### Nrf2 modulates the transcription of some pro-inflammatory genes

**Fig 4:**
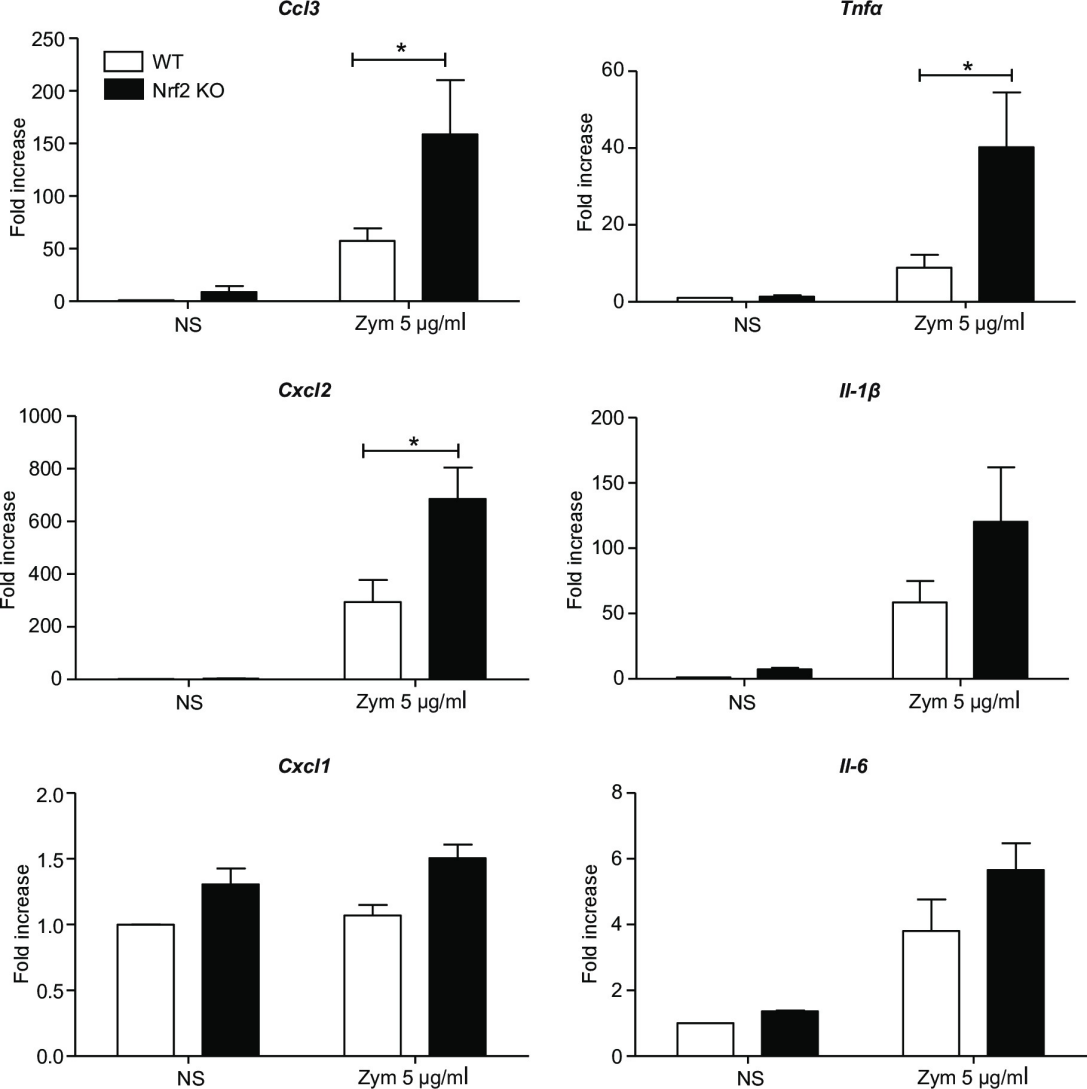
Nrf2 modulates *Ccl3*, *Cxcl2* and *Tnfα* transcription. WT and Nrf2 KO PMNs were incubated or not with zymosan 5 μg/ml for 4 h. mRNA expression of *Ccl3*, *Cxcl2*, *Cxcl1*, *Tnfα*, *Il-1β* and *Il-6*, were measured using RT-qPCR. All results are expressed as fold increase normalized to WT NS and corrected by the expression of the housekeeping genes *β-actin* and *gapdh* (4 independent experiments, n = 4; *p<0.05 Mann-Whitney test).

TNFα, IL-6 and IL-1β are key pro-inflammatory cytokines produced by PMNs. We sought to demonstrate whether Nrf2 could control the transcription of genes encoding these cytokines as well as the chemokines CCL3, CXCL1 and CXCL2. For that, their transcriptional level was quantified in WT and Nrf2 KO PMNs stimulated or not with zymosan 5 μg/ml for 4 h. Interestingly, the lack of Nrf2 allowed for considerable transcription of genes encoding CCL3, CXCL2, TNFα and IL-1β in response to zymosan. As shown in Fig 4, Nrf2 KO PMNs displayed a significantly enhanced *Tnfα*, *Cxcl2* and *Ccl3* mRNA expression as compared to WT PMNs, while *Il-6*, *Cxcl1* and *Il-1β* mRNA expressions were not significantly increased in the absence of Nrf2.

### Nrf2 is not involved in the phagocytic capacity of PMNs

**Fig 5:**
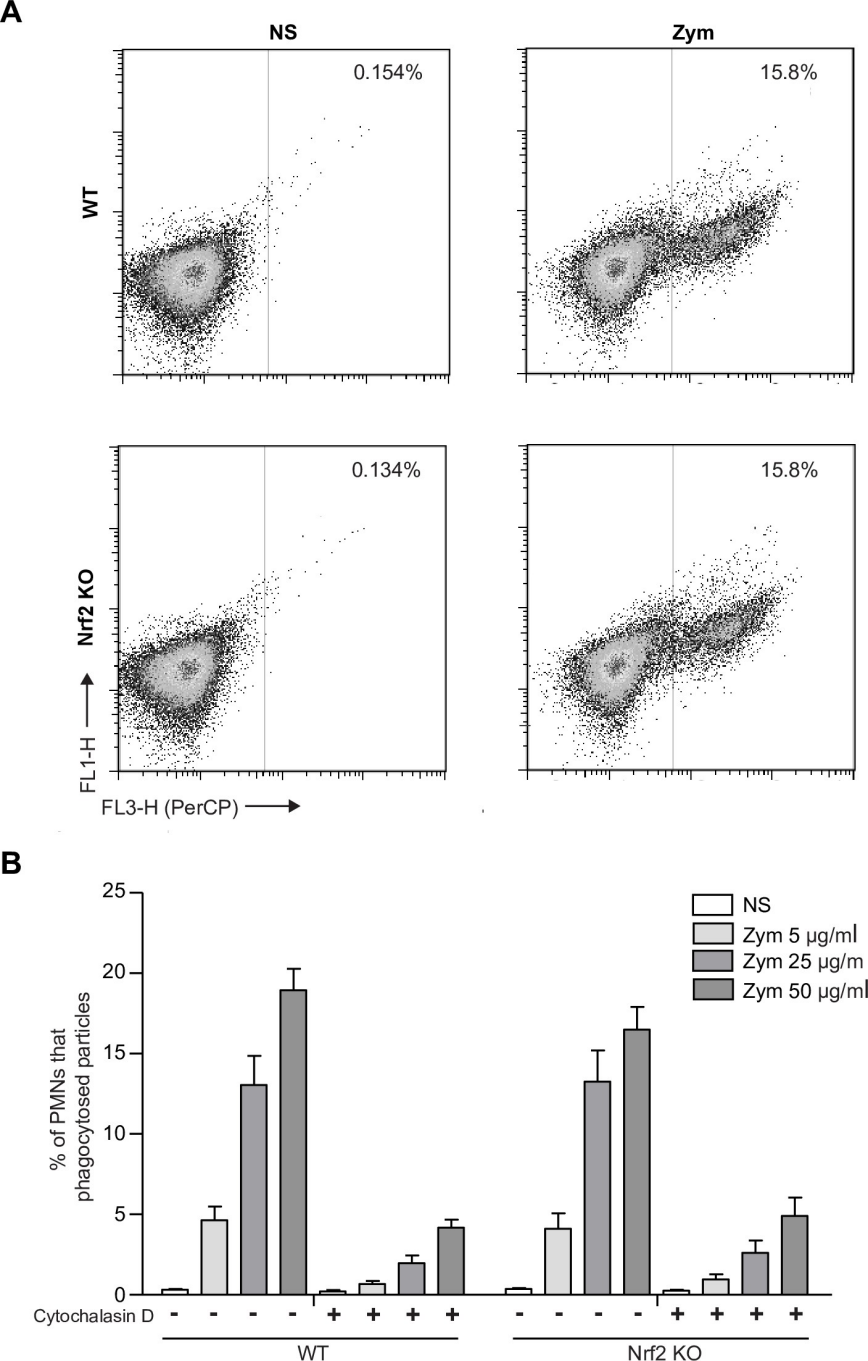
The lack of Nrf2 does not affect PMN phagocytic capacity. Cells were incubated for 90 min alone or with the determined concentrations of pHRodo Red Zymosan Bioparticles. (A) Flow cytometry-based analysis was used to evaluate PMN zymosan uptake. PMNs were subjected to a one-color analysis for the percent of zymosan (25 μg/ml) positive events. (B) Graph representing the percentage of PMNs that phagocytosed increasing concentrations of zymosan (5, 25 and 50 μg/ml). PMNs were pretreated for 30 min with 20 μM of cytochalasin D for negative control. All data are presented as the mean ± SEM, 4 independent experiments, n = 4.

We then compared the phagocytic capacity between WT and Nrf2 KO PMNs. This function was analyzed using pHrodo Red Zymosan Bioparticles Conjugates. We found that WT and Nrf2 KO PMNs showed similar capacity to phagocyte zymosan bioparticles after 90 min, in a concentration-dependent manner. Similarly, the inhibition effect of Cytochalasin D (inhibitor of actin polymerization) was similar in both genotypes (Fig 5A and 5B). These data suggest that Nrf2 does not seem to be involved in the phagocytosis of zymosan particles by PMNs.

### Nrf2 is required for an optimal PMN migration independently of chemokine receptor expression and ATP level

**Fig 6:**
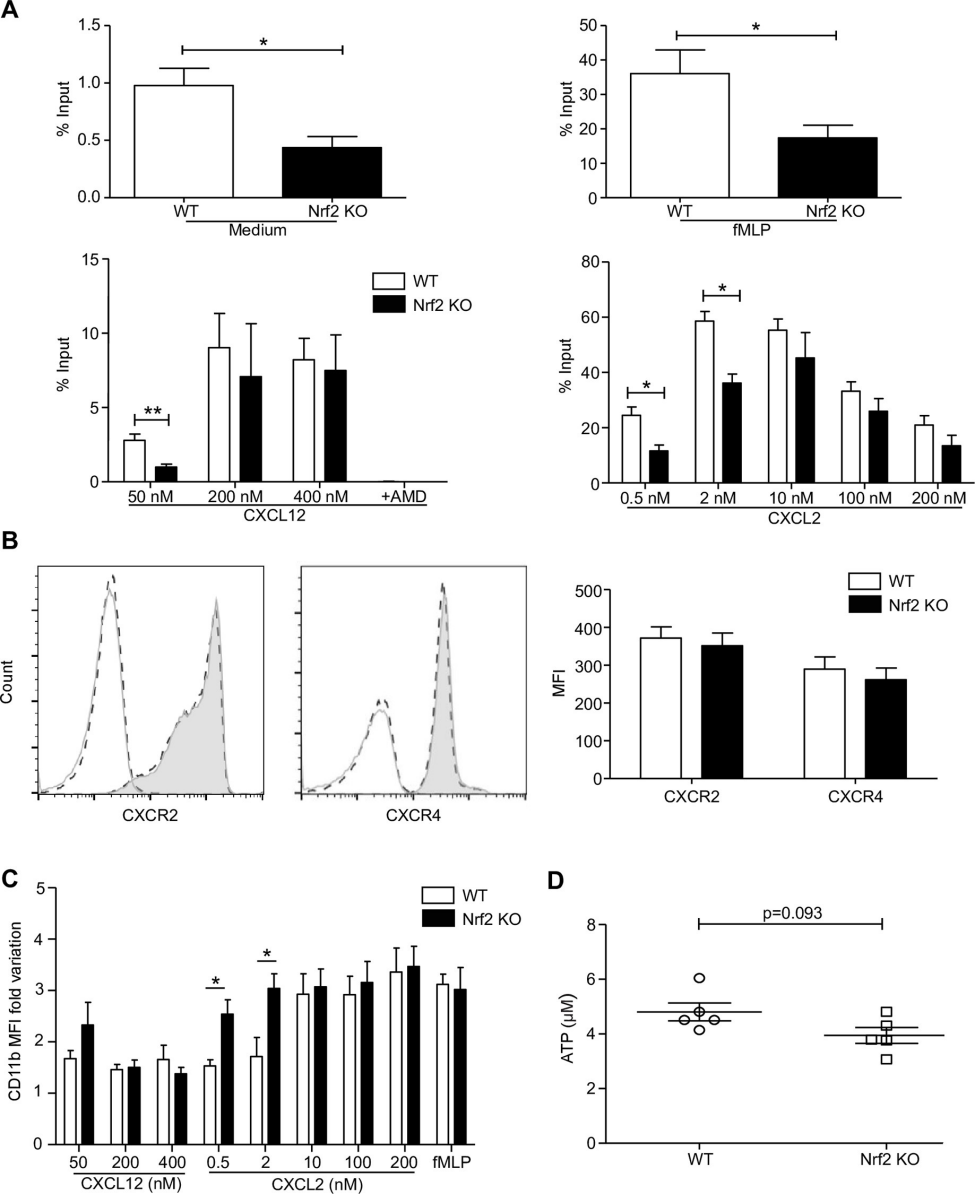
Nrf2 is necessary for optimal PMN migration toward CXCL12 and CXCL2 without regulating receptors expression. (A) WT and Nrf2 KO PMNs were allowed to migrate for 1 h across transwell filters spontaneously or toward fMLP 1 μM, CXCL12 or CXCL2. The negative control in CXCL12-dependent migration was obtained by pretreating PMNs with 50 μM of AMD300. Chemotactic indexes were then calculated by dividing the number of PMNs that were counted in the chemokine-stimulated well by the number of PMNs that were counted in the input well (*p<0.05 **p<0.01 Mann-Whitney test). (B) The expression of CXCR2 (B, left panel) and CXCR4 (B, right panel) were assessed on freshly isolated WT PMNs (solid grey line) and Nrf2 KO PMNs (dashed black line) in comparison to corresponding isotypes (unfilled curves). The associated graph represents the mean ± SEM of CXCR2 and CXCR4 MFI, n = 4. (C) The MFI fold variation of CD11b was assessed on PMNs that have migrated through the transwell in comparison to PMNs in the input well. (D) ATP levels in freshly isolated PMNs were quantified using an ATP luminescent assay. The expression levels from 6 independent experiments are shown as the mean of MFI ± SEM (n = 6).

We finally studied the role of Nrf2 in the modulation of PMN migration. We focused on two axes (CXCR2 and CXCR4) because *in vivo* PMN recruitment relies heavily on their ligands CXCL2 and CXCL12 respectively; fMLP was used as a positive control. Using a transwell migration assay, PMNs were allowed to migrate for 1 h spontaneously, toward 1 μM of fMLP or toward gradual concentrations of CXCL2 and CXCL12. In the absence of Nrf2, PMN spontaneous migration and migration in response to fMLP were significantly decreased as compared with WT PMN migration. Concerning the response to increasing concentrations of CXCL12, both WT and Nrf2 KO PMNs displayed a relatively weak capacity to migrate. Interestingly, Nrf2 KO PMNs displayed a significantly decreased capacity to migrate in response to the lowest concentration of CXCL12 as compared to WT PMNs. This migration was completely abolished in the presence of AMD, a competitive inhibitor of CXCL12. Concerning the dose-response of CXCL2, two distinct bell curves could be observed, one shifted in relation to the other. Indeed, Nrf2 KO PMNs displayed a significantly decreased capacity to migrate in response to the two lower concentrations of CXCL2, while migration in response to higher concentrations was similar in both WT and Nrf2 KO PMNs (Fig 6A). These results suggest that Nrf2 could participate in PMN motility by increasing their sensitivity to chemoattractants.

In order to assess whether these differences in migration in the absence of Nrf2 could be linked to differences in the expression of chemokine receptors, we analyzed CXCR4 and CXCR2 membrane expression on freshly isolated PMNs. As observed, WT and Nrf2 KO PMNs displayed similar expression of both receptors (Fig 6B), suggesting that Nrf2 can participate in PMN migration independently of chemokine receptor expression.

In order to better understand the mechanism of Nrf2 KO PMN affected migration, we analyzed the PMN phenotype after 1 h of migration by quantifying the expression of the β2 integrin CD11b/CD18 adhesion molecule. We found that the migration-induced upregulation of CD11b was significantly increased in Nrf2 KO PMNs as compared with WT PMNs, in response to low concentrations of CXCL2 (Fig 6C).

Finally, as ATP is mandatory for an optimized migration we hypothesized that ATP levels could differ in Nrf2 KO PMNs. We found no significant difference between both genotypes at resting state, even if ATP levels seemed to be slightly higher in WT PMNs (Fig 6D).

## Discussion

PMNs play a key role in host defense against pathogens, but inadequate or excessive activation can lead to deleterious effects, contributing to the pathophysiology of many acute and chronic inflammatory diseases. In parallel, Nrf2 plays an active role in the control of inflammation, via several mechanisms. In this study, using an *in vitro* model, we found that Nrf2 participates in the regulation of murine BM-derived PMN response to the fungal stimulus zymosan, *via* the activation of cytoprotective genes and the downregulation of pro-inflammatory genes. ROS production depends on Nrf2 activation, while phagocytosis is similar in WT and Nrf2 KO mice PMNs. Our results thus highlight the impact of Nrf2 on PMN activation, providing new findings relevant to the regulation of neutrophil activation by zymosan.

Several studies have led to the consensus that PMNs are able to finely regulate both innate and adaptive immune responses, engaging with T lymphocytes or antigen-presenting cells, releasing anti-inflammatory mediators or expressing regulatory receptors; this broader role in immunity also led to the definition of several functional PMN subpopulations [4,39–41]. In particular, our group evidenced that isolated NETs or neutrophil-derived ectosomes were able to downregulate LPS-induced DC maturation and their capacity to induce T lymphocyte proliferation [7,42]. We also evidenced that GILZ, a potent anti-inflammatory mediator implicated in cell survival, was present in human neutrophils, promoted apoptosis *via* Mcl-1 downregulation, and was upregulated in patients with the acute respiratory distress syndrome in relation to severity [11,43]. Our group is also very involved in studying the role of Nrf2 in several pathophysiological conditions. For instance, we showed that Nrf2 can control inflammation in a model of allergic contact dermatitis (both sensitization and effector phases) [44] and during sclerodermia [21]. We also highlighted the role of Nrf2 in the control of DC cell death induced by chemical sensitizers and we found that Nrf2 positively controls antioxidant genes like *gsr*, *catalase*, *gpx*, *nos2* and some immune genes in response to contact sensitizers [19]. We thus addressed the question of the potential immunoregulatory role of Nrf2 in numerous PMN functions.

Our first aim was to study Nrf2 activation in BM-derived PMNs. Joshi *et al*. recently demonstrated that the transcription level of Nrf2 was strong in circulating PMNs and in wound tissue PMNs [32]. Here, using flow cytometry, we confirm that Nrf2 is highly expressed in BM-derived WT PMNs as compared to other BM-derived cells. Moreover, using SFN, a well-characterized Nrf2 activator, we demonstrate that Nrf2 can be mobilized and activated in WT PMNs as several target genes were induced; SFN was particularly potent for the activation of *Nqo1* and *Cat*. In addition, our study evidences for the first time that zymosan, a TLR2 agonist is also able to mobilize Nrf2 in WT PMNs and to activate the same target genes. These results showing the important activity of Nrf2 in PMNs are consistent with those of Joshi *et al*. in mouse blood PMNs [32], and with those of R Thimmulappa *et al*. in human PMNs [30] and VC Araujo *et al*. in human PMNs from fungal oral granuloma [45].

Since Keap1/Nrf2 signaling maintains redox homeostasis in the cell, we studied the role of Nrf2 in PMN oxidative burst. We thus performed a set of experiments aiming to document the contribution of Nrf2 in ROS production. C Sima *et al*. have recently reported that in response to PMA, Nrf2 KO PMNs displayed a normal ROS production [31]. Conversely, an enhanced ROS release in Nrf2 KO PMNs has been described in the literature in response to LPS; however, experimental conditions were quite different as PMNs were collected from the peritoneal fluid 4 h after thioglycolate injection [24]. In our study, we were able to detect a low but significant Nrf2-mediated regulation of ROS production in response to zymosan, independently of TLR2 and dectin-1 expression level. This suggests that Nrf2 could rather intervene in the downstream signaling of these receptors. We chose non opsonized zymosan as a stimulus as it has been demonstrated that it induces NADPH oxidase activation in human blood PMNs leading to high ROS production [33]. Since zymosan induces Nrf2 accumulation, the antagonistic activities of Nrf2 and NADPH oxidase [27] could explain the exacerbated zymosan-induced ROS production in the absence of Nrf2. Our second set of experiments was designed to greatly upregulate Nrf2 before stimulating oxidative burst. We indeed found that SFN pretreatment reduced zymosan-induced ROS to a greater extent in WT PMNs than in Nrf2 KO PMNs, suggesting that Nrf2 has a crucial role in ROS regulation in BM-derived PMNs. Of note, we only used here low concentrations of SFN to avoid activation of other signaling pathways [46]. The latter results are consistent with those of Thimmulappa *et al*. using the triterpenoid CDDO-Im as Nrf2 enhancer or those of Dias *et al*. using SFN in human PMNs [47,48]. Using live cell imaging of brain hippocampal glio-neuronal cultures, other authors also found that a graded expression of Nrf2 paralleled a graded production of ROS [27].

Several studies have documented the Nrf2-induced decrease in transcriptional expression of some pro-inflammatory cytokines in various cell types, such as human epithelial cells or macrophages [49,50]. In PMNs, few results have been reported. Here we have clearly shown that, in response to zymosan, Nrf2 KO PMNs displayed a significantly increased *Ccl3*, *Cxcl2* and *Tnfα* mRNA expression. This suggests that Nrf2 can modulate inflammatory gene transcription in BM-derived mice PMNs, as already suggested in peritoneal PMNs in a model of LPS-induced inflammation [30] and in human blood PMNs [47]. Moreover, we would suggest that Nrf2 activation in zymosan-induced peritonitis could limit neutrophil activation and auto-recruitment mainly *via* the regulation of TNFα, CXCL2 and CCL3 production in the peritoneal fluid [51]. Concerning IL-6, WT and Nrf2 KO PMNs exhibited similar transcript levels, in accordance with N Joshi *et al*. recent study [32]. Although Nrf2 decreases the transcriptional upregulation of *Il-6* and *Il-1β* in LPS-stimulated macrophages through the inhibition of RNA Pol II recruitment, no direct interference could be observed in PMNs [25]. Interestingly, it was also reported that Nrf2 and its target genes were not needed for interferon γ production by lung PMNs in a mouse model of pneumonia [52]. We can thus suggest that, Nrf2 is needed for the transcriptional regulation of several but not all inflammatory cytokines and chemokines in PMNs.

We also examined whether the lack of Nrf2 can affect PMN capacity to phagocyte zymosan particles. We found that Nrf2 KO and WT PMNs exhibited similar capacity to phagocyte zymosan bioparticles. While Nrf2 KO peritoneal macrophages have been described to exhibit impaired phagocytosis during sepsis as compared with WT cells [24], our results suggest that Nrf2 is not involved in PMN phagocytosis, unlike macrophages. This result highlights several specific roles of Nrf2 depending on cell type.

The role of ROS in PMN migration has been widely studied over the past few years [53,54]. A potential link between Nrf2 and PMN migration can thus be suspected. Although some studies have shown an enhanced PMN recruitment in Nrf2 KO mice in different inflammatory settings [55,56], the use of a full Nrf2 KO model could not lead to clear conclusions concerning Nrf2 role in PMN migration; in particular, Sima *et al*. suggested that Nrf2 could slow down fMLP-induced PMN migration *in vitro* as early as 15 min of migration [57]. We thus further investigate this mechanism, using an one hour-transwell migration assay with fMLP and increasing concentrations of two other chemokines, CXCL2 and CXCL12 that antagonistically modulate PMN chemotaxis [58,59]. In contrast with ROS shifted curves, the chemokine bell-shaped migration curves suggested that Nrf2 improved PMN ability to migrate toward fMLP or low concentrations of CXCL2 and CXCL12. Of note, WT and Nrf2 KO PMNs displayed a similar expression of CXCR2 and CXCR4. Nrf2 has been previously reported to partially control hematopoiesis through the regulation of CXCR4 signaling [60]. Here, we saw that Nrf2 could also regulate PMN CXCR2- and CXCR4-dependent migration independently from the expression level of both receptors. To better understand the mechanism of Nrf2-modulated migration, we compared the modification of expression of β2 integrin CD11b/CD18 after 1 h of migration and found that Nrf2 KO PMNs exhibit a significant upregulation of CD11b even in response to low concentrations of CXCL2. CD11b upregulation on *in vitro*-migrating PMNs has already been described, in particular in a recent model of airway epithelial cells [61]. This suggests that Nrf2 ensures an optimal migration of PMNs via the control of migrating cell activation level. Some studies have evidenced that Nrf2 could potentially enhance the synthesis of ATP in various cell models [62,63]. As ATP is crucial for PMN migration [64], we thus hypothesized that ATP could be involved in the modified migration observed in Nrf2 KO PMNs. However, we failed to evidence a significant difference between ATP levels in resting WT and Nrf2 KO PMNs suggesting that Nrf2 plays a weak role in this setting. Based on our findings, we can suggest that the intense recruitment of PMNs observed in Nrf2 KO mice in several inflammatory *in vivo* models [55,56] might not be related to an intrinsic control of PMN migration by Nrf2. Nevertheless, Nrf2 could ensure an optimal PMN motility and chemokine sensing needed, in particular to drive PMN migration from the BM to the tissues at the very early stages of inflammation.

This *in vitro* evaluation of Nrf2 implication in PMN functions need also to be analyzed from a clinical point of view as Nrf2 diseasome and drugome have been recently defined [34]. Indeed, in the numerous clinical settings associated with significant PMN tissue infiltration and/or PMN activation, it is important to better understand the implication and the efficiency of regulatory mechanisms including Nrf2 pathway. For instance, in the lung, activating Nrf2 leads to protective effects during acute lung injury or asthma, but also enhances advanced stages of carcinogenesis [65,66]. We can thus assume that activating or inhibiting Nrf2 in PMN-associated diseases is an important issue that needs to be fully considered.

Taken together, our results contribute to a better understanding of the Nrf2 protective role in PMNs, particularly in response to zymosan that is a physiological stimulus mimicking yeast infection, and widely used in experimental models of arthritis or peritonitis. This study indicates that Nrf2 cytoprotective target genes are inducible in PMNs and highlights its oxidative burst regulation capacity. In addition, this study demonstrates that Nrf2 is needed for an optimal PMN migration. Future research into understanding the role of Nrf2 in PMN recruitment in a context of inflammation may provide insight and novel approaches in the field of inflammation.

## Supporting information

S1 Fig Flow cytometry-based assessment of PMN purity. Following negative isolation, PMN purity was assessed using antibodies against Ly6G and CD11b. Ly6G^+^, CD11b^+^ cells represent BM PMN. PMN purity was > 95% in all experiments.
(TIF)

S2 Fig Assessment of SFN toxicity through AnnexinV-7AAD staining. PMN were incubated alone or with the indicated concentrations of SFN for 4 h, and then stained with AnnexinV and 7-AAD. AnnV^+^ cells represent apoptotic cells while double positive cells (AnnV^+^,7-AAD^+^) represent necrotic cells. Data are shown as representative FACS analysis (A) and as the mean ± SEM of 3 independent experiments (B).
(TIF)

S3 Fig Zymosan fails to induce DNA release in netosis. PMN were incubated for 3 h, alone or with PMA 100 nM and zymosan 50 μg/ml. Time dependent DNA release was monitored using the fluorescent SYTOXgreen. (A) Data from one representative experiment shows the kinetic of DNA release in response to PMA 100 nM. (B) Results from 4 independent experiments are expressed as the difference between RFU at time 15 min and the RFU at time 180 min.
(TIF)

## Abbreviations

BM: Bone marrow
DC: Dendritic cell
GILZ: Glucocorticoïd-induced leucine zipper
GPX: Glutathione peroxidase
HMOX-1: Heme oxygenase-1
IL-: Interleukin
KEAP 1: Kelch-like ECH-associated protein 1
MPO: Myeloperoxidase
NET: Neutrophil extracellular traps
NOX2: NADPH oxidase 2
NQO1: NAD(P)H quinone oxidoreductase 1
NRF2: Nuclear factor (erythroïd-derived 2)-like 2
PMNs: Polymorphonuclear neutrophils
ROS: Reactive oxygen species
SFN: Sulforaphane
TLR: Toll-like receptor
TNFα: Tumor necrosis factor α
