# Supplementary figures and images for: Nrf2 downregulates zymosan-induced neutrophil activation and modulates migration
Source: PLoS One. 2019 Aug 16;14(8):e0216465. doi: 10.1371/journal.pone.0216465 (PMC6697320; doi:10.1371/journal.pone.0216465)

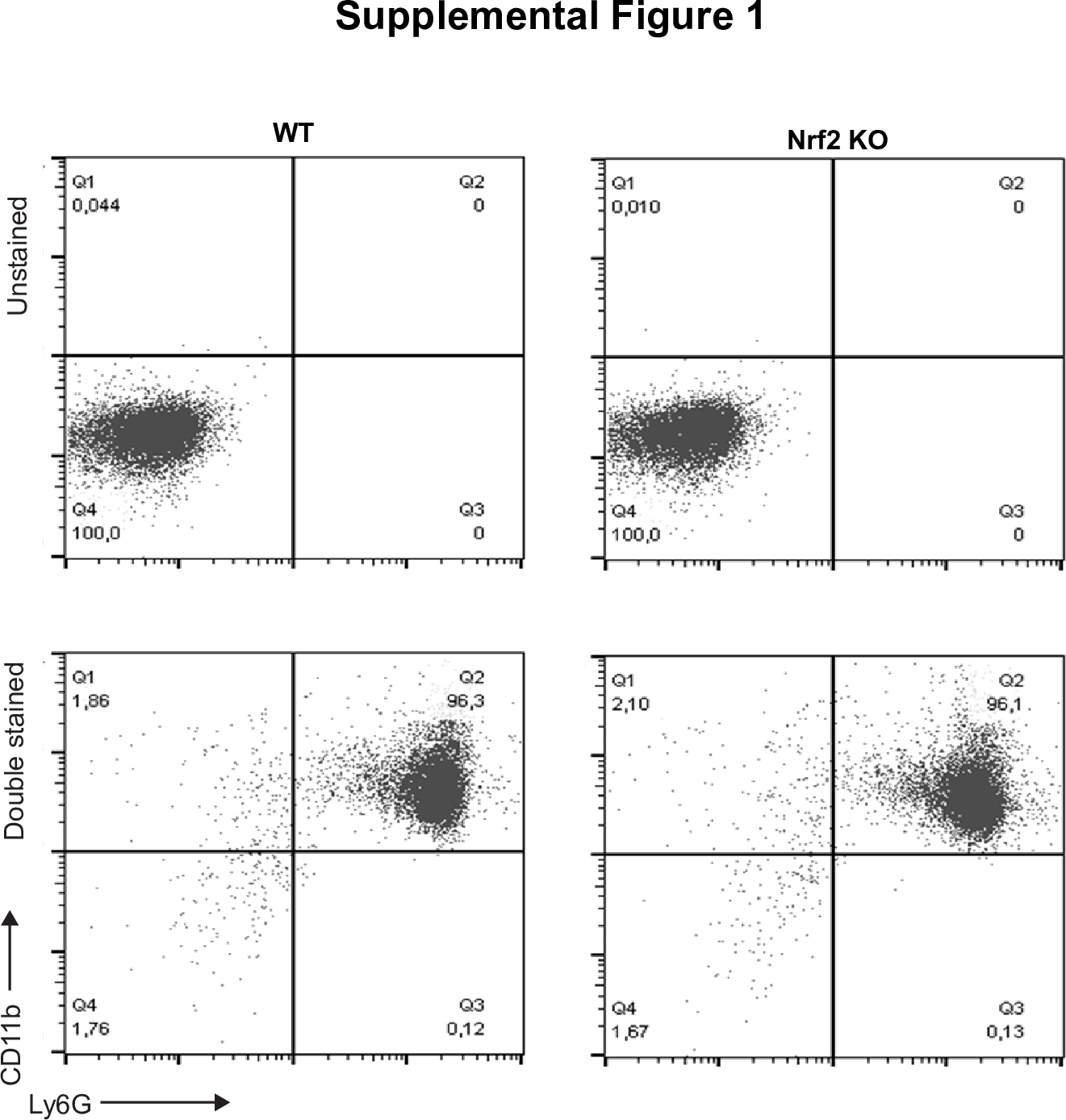

Supplement: S1 Fig — Following negative isolation, PMN purity was assessed using antibodies against Ly6G and CD11b. Ly6G+, CD11b+ cells represent BM PMN. PMN purity was > 95% in all experiments. (TIF) [file pone.0216465.s001.tif]

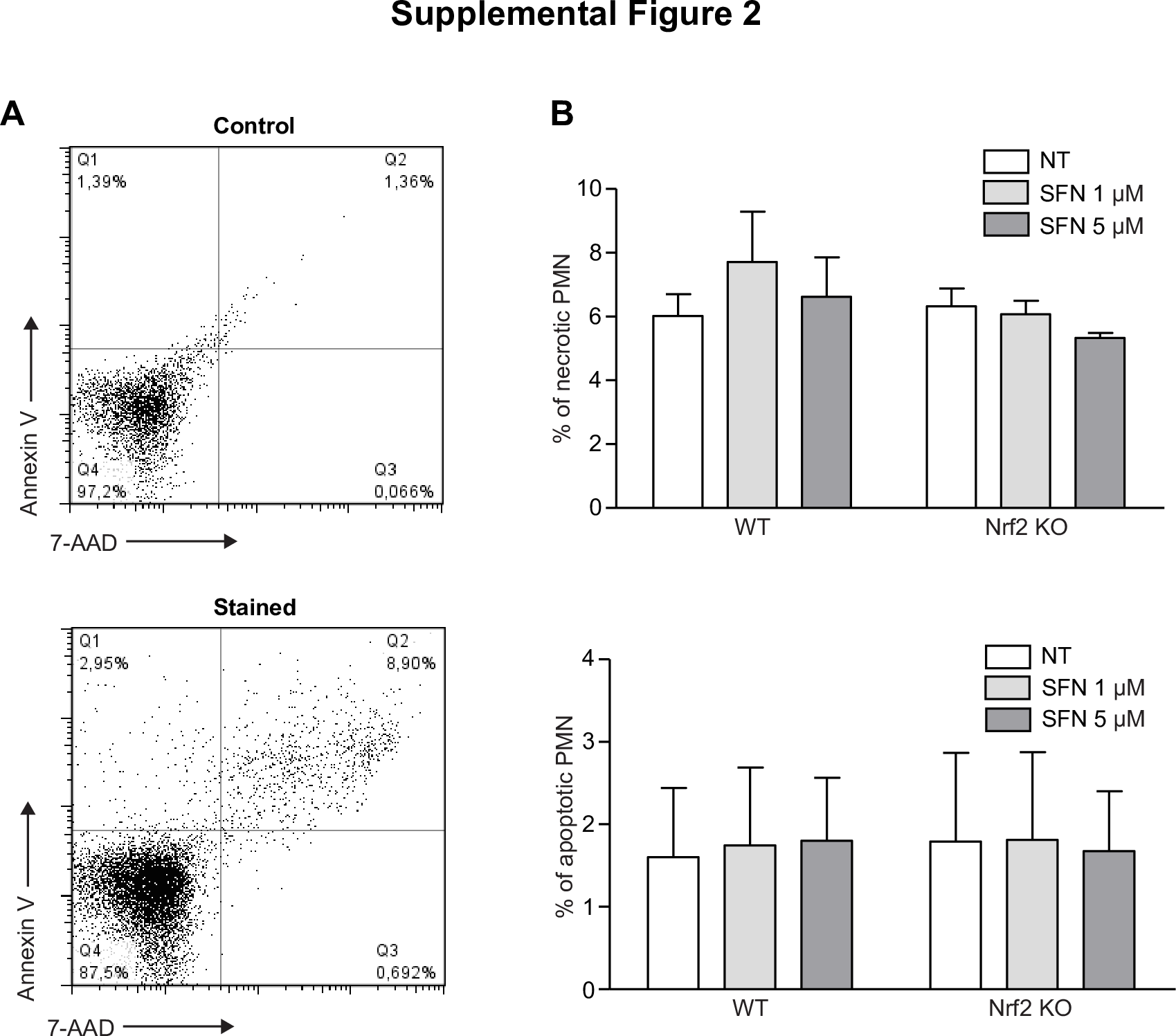

Supplement: S2 Fig — PMN were incubated alone or with the indicated concentrations of SFN for 4 h, and then stained with AnnexinV and 7-AAD. AnnV+ cells represent apoptotic cells while double positive cells (AnnV+,7-AAD+) represent necrotic cells. Data are shown as representative FACS analysis (A) and as the mean ± SEM of 3 independent experiments (B). (TIF) [file pone.0216465.s002.tif]

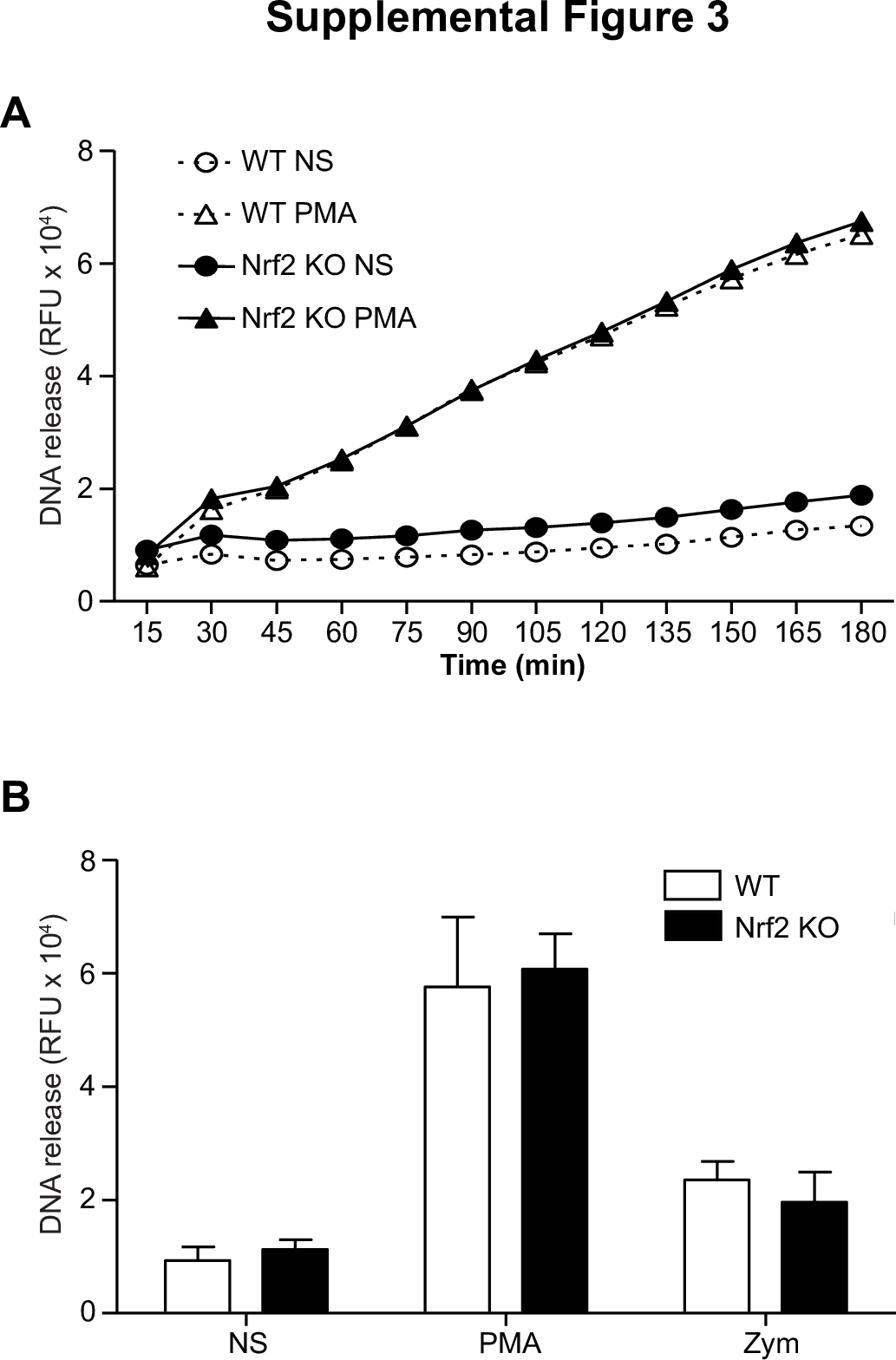

Supplement: S3 Fig — PMN were incubated for 3 h, alone or with PMA 100 nM and zymosan 50 μg/ml. Time dependent DNA release was monitored using the fluorescent SYTOXgreen. (A) Data from one representative experiment shows the kinetic of DNA release in response to PMA 100 nM. (B) Results from 4 independent experiments are expressed as the difference between RFU at time 15 min and the RFU at time 180 min. (TIF) [file pone.0216465.s003.tif]
